# Supplementary suckling technique in infants less than 6 months of age with uncomplicated severe acute malnutrition: a prospective hospital-based study in armed conflict Yemen

**DOI:** 10.1186/s12887-022-03745-w

**Published:** 2022-11-21

**Authors:** Mohamed Salem Mohamed Baazab, Jalal Ali Bilal, Iman Ali Ba-Saddik, Ali Mohammed Arabi

**Affiliations:** 1 Faculty of Medicine and Health Sciences, University of Aden, Aden, Yemen; 2 Pediatrics Department, College of Medicine, Shaqra University of Shaqra, Shaqra, Saudi Arabia; 3 Faculty of Medicine, University of Khartoum, Khartoum, Sudan

**Keywords:** Severe acute malnutrition, Infants supplementary suckling outcome, Armed conflict Yemen

## Abstract

**Background:**

Globally 3.8 million of children under 6 month of age are severely wasted. In Yemen, around 20% of children under 6 months were affected by malnutrition during the armed conflict in the last 7 years. Supplementary suckling may reestablish exclusive breastfeeding in infant less than 6 months of age with Severe Acute Malnutrition (SAM). This study aimed to determine the outcomes of employing supplementary suckling technique in treatment of uncomplicated SAM infants in a conflict-affected community.

**Methods:**

A prospective hospital-based study was carried out between January to April 19th, 2020 among randomly selected infants less than 6 months of age with SAM following breastfeeding failure. Infants’ anthropometric indices were daily measured and recorded. Supplementary sulking technique was used in management with high or low protein milk-based formula supplement. Outcome was recorded as cured, died, defaulter or in nonrecovery state.

**Results:**

In this study 108 infants were enrolled with a median (IQR) age of 4 (2.5-5) years and a male: female ratio of 1.4:1. After treatment, 80.6% recovered to cure, 12% defaulters, 6% died, and 2% did not respond to treatment Thirty-four infants (38.8%) gained weight with significantly increased median weight and median weight-for-age z score. The median (IQR) duration of treatment was 9 (7.5-14) days. The means of age and weight-for-length z score were correlated (*r* = − 0.22, *p* = 0.025). Duration of treatment was a predictor of outcome (OR = 1.71, 95% CI = 0.05-0.62, *p* < 0.001).

**Conclusion:**

Supplementary suckling technique for feeding infant with SAM aged less than 6 months had a positive impact on anthropometric indices with high cure rate. The younger the infant and the longer the duration of treatment, the better the outcome.

**Supplementary Information:**

The online version contains supplementary material available at 10.1186/s12887-022-03745-w.

## Background

Globally, undernutrition in all its forms is still a challenge even without accounting for the impact of the COVID-19 pandemic in 2020 [1]. Wasting in children is the serious outcome of poor nutrient intake and/or disease as it weakens immunity and delays development especially when wasting is severe. In 2020,45.4 million (6.7%) children under 5 years of age were affected by wasting, of which 13.6 million were severely wasted [2]. Kerac et al reported that globally 3.8 million of children less than 6 month of age are severely wasted [3].

The first 1000 days’ period is the time from conception through approximately 2 years of age. Nutritionists have recently stressed on the “first 1000 days” as a golden opportunity to influence the child outcome with short- and long-term effects on the health of the infants and young children. Environmental factors and nutrition during this period can have positive effects on a baby’s growth, brain development, digestive tract, metabolism and immune system. The first 6 months of life is crucial for optimum brain and overall development in late fetal and early postnatal life. Hence, early supplementation of nutrients especially during the first 6 month of life by breastfeeding improves neurodevelopmental outcome over extended periods of life is advocated for all nutrition programs guidelines [4, 5].

Yemen is the largest humanitarian crisis and it has been overwhelmed by one of the world’s worst food crises, with nearly 2.3 million children under the age of five suffer from acute malnutrition in 2021 [6]. Twenty percent of children under 6 months were affected by malnutrition during the armed conflict in Yemen and 11.5% had acute severe malnutrition (SAM). Exclusive breastfeeding rate among Yemeni infants less than 6 months of age was 43%, well below the worldwide rate probably because conflict intensity is negatively associated with breastfeeding incidence [7, 8].

The controversy of feeding approaches for infants less than 6 months of age could not enable policy makers to recommend a solid unified recommendation for feeding of these infants [9]. However, feeding support aims to establish effective exclusive breastfeeding as the mainstay of treatment. Lactation was successfully reestablished after breastfeeding failure using supplementary suckling technique (SST) [10].

Supplementary suckling technique is used in infants less than 6 months of age suffering from SAM, emphasizing the support and sustain of breastfeeding through counseling and re-establishment of lactation in mothers with lactation failure. SST provides infant less than 6 months of age with SAM with therapeutic milk to initiate rehabilitation and weight gain, besides aiming to reestablish exclusive breastfeeding through stimulating relactation [10]. Despite SST is being recommended in SAM management in young infants [9, 11], studies on SST for treatment of uncomplicated SAM in infants, particularly in disastrous regions is dearth. This study aimed to determine the outcome of employing SST in treatment of uncomplicated SAM infants in a conflict-affected community. SST is often mentioned for treatment of SAM but opinions differ as great success was reported as well as ineffectiveness. However, its effectiveness in war-conflict setting was not satisfactorily assessed. Feeding on SST in war-conflict settings could enable young infants who develop SAM following lactation failure to gain satisfactory weight.

## Methods

### Study type, population and duration

This was a prospective hospital-based study among infants less than 6 months of age with SAM, following breastfeeding failure. The study was conducted during the period between January 1st, 2018 through April 19th, 2020 in both domains.

### Settings

This study was conducted in the Paediatric Department at Al-Sadaqa Teaching Hospital in Sheikh Othman district, Aden, Yemen. It is a tertiary referral hospital, which serves all referral cases from surrounding provinces of Aden city. The treatment feeding centre (TFC) is located inside the hospital of 21 beds with 4 doctors and 15 nurses. Bed occupancy is 3 patients per month and the mortality rate is < 5% per year.

### Sample size, type and data collection

Badi et al. has documented the prevalence of malnutrition in Aden as 5.2% among hospitalized children [12]. The sample size (n) was calculated using open OpenEpi, Version 3, open-source calculator—SSPropor where the hospitalized population was approximately 12,000 children within a confidence interval of 97% and a design effect of 1 and yielded a sample of 97. In this study a total of 108 infants were included.

### Inclusion and exclusion criteria

Inclusion criteria were infants less than 6 month of age with SAM due to failure of breastfeeding who were admitted for feeding in TFCs. SAM was diagnosed based on the definition of the updated WHO criteria for management of SAM in infants and young children [9]. These were weight-for-length less than − 3 Z-score, or the presence of bilateral pitting oedema. In addition, all mothers were unable to successful breastfeeding at admission due to failure of lactation owing to stress of arm-conflict.

Infants with known underlying organic diseases as a cause of malnutrition were excluded. Any infant with a known congenital, chronic or metabolic disease as a cause of SAM was excluded. Furthermore, an infant was excluded if he or she had any serious clinical condition or medical complication, recent weight loss, ineffective feeding (attachment, positioning and suckling) directly observed for 15–20 min, any medical or social issue needing more detailed assessment or intensive support (e.g., disability, depression of the caregiver, or other adverse social circumstances) [9].

Infants were chosen randomly using a computer-generated set of random numbers. Random procedure was applied ensure that every infant had an equal chance of selection.

A validated questionnaire containing personal and clinical data was filled and labelled with a code number.

### Study technique

All infants included in the study were assessed and evaluated clinically. Data of the main socio-demographic characteristics and clinical attributers were recorded using a data collection sheet. The infants were weighed to the nearest decimal point on daily basis using a beam balance scale. Length was initially measured and then every 5 days thereafter. Bilateral lower limb pitting edema was confirmed by pressing on the dorsum of the lower limbs [13]. Weight was measured using SECA® 384 Electronic Baby/Toddler Scale and length was taken for infant while lying down using the UNICEF wooden portable baby/child length/height measuring board.

**Fig. 1:**
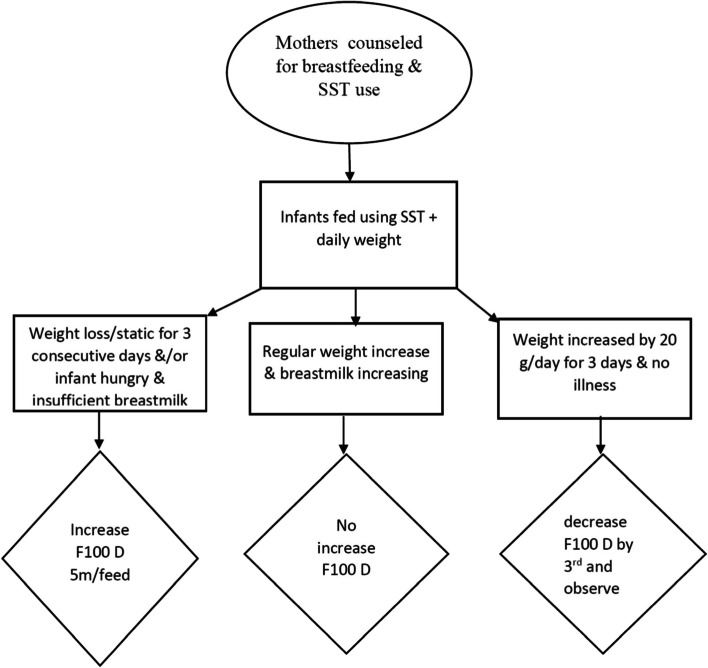
Protocol outline of supplementary suckling technique used for infants with SAM in the study

Supplementary suckling technique (SST) was used in the management of SAM infants using supplementary feeding formulas Diluted F feeding (DF100), a milk formula with higher protein and energy content with cup for those presenting with no oedema, while on the other hand those with oedema were fed on feeding formula F75, a low-protein milk-based formula diet. All mothers were counseled about breastfeeding attachment and positioning according to the WHO guidelines [14]. Mothers were then trained on the use of SST in TFC. Nasogastric tube size 8 was used for feeding. The tip of the tube was cut about 1 cm and the end of the tube was put in a cup with supplementary diluted F100 or F75. The tip of the tube adhered to the nipple was put inside the angle of the infant’s mouth during breastfeeding. The cup was initially put 5- 10 cm below the breast, while the child was breastfeeding, the milk from the cup was sucked up through the tube and was taken by the infant initially. The cup was then gradually lowered to approximately 30 cm below the level of the breast so that the milk did not flow too quickly. The volume of F100D in the cup was gradually reduced when the infant gained weight for 2-3 days (at least 20 g per day), was free from illness and breastmilk flow was evident. Then supplementary milk in the cup (F100D) was reduced by one third and the mother continued breastfeeding for 2 or 3 days. When the weight continued to rise, the amount of supplementary milk (F100 D) was reduced until the milk was no longer needed and the infant showed gain in weight from exclusive breastfeeding without any supplementary milk. If weight gain was not satisfactory when the volume of supplementary milk was reduced then the policy was to increase the volume to the previous level for the next 2 days, with a successive repeat trial. Infants are fed on F100 D in a volume of 130 ml/kg body weight per day. This volume was increased 5 ml/feed if the child had lost weight or had static weight for 3 consecutive days or was continuing to be hungry after taking all his feeds. The outline of the protocol used in the treatment of infants in this study is depicted in Fig. 1.

All infants received a course of antibiotic with amoxicillin. Four infants developed signs of pneumonia and three had gastroenteritis and sepsis who were all then consequently transferred to the inpatient care and received all necessary treatment but succumbed thereafter.

An infant was discharged if he/she was gaining adequate weight on isolated breastfeeding to be followed in the nutritional units at health centers.

### Outcome variables

These denoted that that infant was cured, died, defaulter or in non-recovery state. Cured denoted an infant who was gaining adequate weight on breastfeeding at the time of discharge. Infants who died were those who were transferred to inpatient care after development of complications at TFC.

The standard indices of nutritional status of children were weight, length, weight-for-length Z score (WLZ) and weight-for-age Z score. Indicators were expressed as Z scores in standard deviation (SD) from the median of the reference population. The WHO Multicentre Growth Reference Study Group growth standards was the reference [15].

### Ethical approval

The Ministry of Public Health and Population in Yemen and the Faculty of Medicine and Health Sciences, University of Aden, Yemen both provided the ethical approval. Mothers agreed to participate in the study after signing an informed consent form.

### Statistics handling

The data collected and the variable results were entered into the computer, using SPSS® (Statistical Package for Social ScienceSPSS Version 24) software for windows. Test of normality (Shapiro-Wilk) was performed on the continuous data and parametric tests were used where the data were normally distributed otherwise nonparametric were used. Continuous variables were expressed as mean (SD) when normally distributed or otherwise median interquartile range (IQR). Hence either the paired student *t* test or the Wisconsin test was used for comparison of two sets of continuous variables. Dichotomous or otherwise nominal variables were displayed as frequency (%). Chi-square test was used to establish association between dichotomous variables.

A binary logistic regression model was employed to determine whether the independent covariates (age, sex, vomiting, diarrhea, anemia, pneumonia, seizure, WAZ scores on admission and discharge and duration of treatment) had any effect on the dependant variable “outcome” as dichotomous variable. The outcome was divided into cured (the infant was gaining weight) and not cured (defaulter, non-recovery or death) and odd ratio (OR) with 95% CI was used for outcome. A *p* value < 0.05 was considered significant.

## Results

**Fig. 2:**
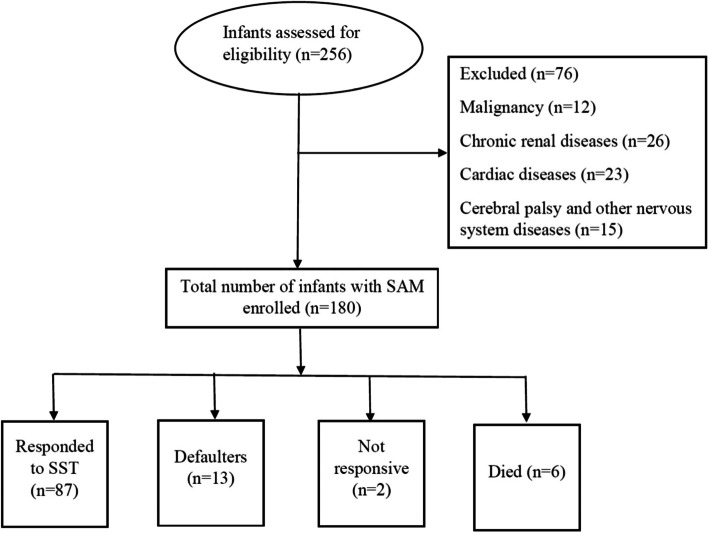
Flowchart of infants with severe acute malnutrition included in the study and the outcome following supplementary suckling technique

The total number of infants included in this study was 108. Number of infants assessed, those who were excluded and the outcome of treated infants is shown in Fig. 2. Their median (IQR) age was 4.0 (2.5) with a range of 4.5 months and a male to female ratio of 1.4:1. All mothers were house-wives and half of them (51%) were illiterates. All infants were clinically wasted and their weight-for-length Z scores were < − 3. The demographic and clinical characteristics are depicted in Table 1.

**Table 1:** Demographic and clinical characteristics of SAM infants (*n* = 108)

Variables	Frequency	%
**Sex**		
Male	63	58.3
Female	45	41.7
**Residence**		
Urban	28	25.9
Rural	80	74.1
**Mother education**		
Illiterate	51	47.2
Primary	43	39.8
Secondary	13	12.0
University	1	0.9
**Water supply and electricity**	90	83.3
**Home displacement**	41	38.0
**Previous hospital admission**	22	20.4
**Vaccination**		
Up-to-date	25	23.1
Partial	41	38.0
Not vaccinated	42	38.9
**Symptoms and signs**		
Pallor	89	82.4
Poor appetite	75	69.4
Normal appetite	31	28.7
Hungry	2	1.9
Diarrhea	70	64.8
Clinical pneumonia	59	54.6
Vomiting	64	40.7
Seizure	3	2.8
Oedema on admission	3	2.8

A highest proportion of all infants, 105 (97.2%) who had no oedema were fed on F100D and SST where only 3 (2.8%) were fed on F75 and SST who were those infants presented with bilateral lower limb oedema.

The higher proportion of the infants (87) with SAM responded to SST treatment accounting for 80.6% cure rate. The defaulters were 13 (12%) and 6 infants died denoting a case fatality rate of 5.6% and only 2 (1.9%) did not respond to treatment hence a non-recovery rate of 1.9%.

Sixty-five (60.2%) infants’ weight-for-length Z scores remained at <− 3 at the time of discharge (including those who succumbed) whereas the rest43 infants (38.8%) gained weight for their weight-for-length Z scores that were between − 3 − + 1 at the same time. In this study, the infants after feeding on SST showed significantly increased median (IQR) weight, increased median weight-for-age Z score. However, there was no significant increase in the median length at discharge compared to that at admission (Table 2). The median (IQR) rate of weight gain during hospital stay was 25.8 (13.4-50) g/Kg/day. This was calculated as the difference between the weights on admission and discharge divided by the hospital stay in days.

**Table 2:** Comparison between nutritional status indices of SAM infants on admission to and discharge from TFC following SST feeding

Variable	Nutritional status indices		***P*** value
	On Admission	On Discharge	
**Weight (grams)**			
**Mean (SD)**	3100 (0.8)	3500 (0.9)	< 0.001
**Median (IQR)**	3000 (2.5-3.8)	3300 (2.800-3.300)	
**Length (cm)**			
**Mean (SD)**	55.4 (5.2)	55.6 (5.2)	
**Median (IQR)**	55 (52.0-59.0)	55 (52.0-59.3)	0.320
**Weight-for-age Z score**			
**Mean (SD)**	−5.2 (1.2)	−4.6 (1.3)	
**Median (IQR)**	−5.4 (− 5.9-4.4)	−4.7 (− 5.3-3.7)	< 0.001
**Weight-for-length Z score Frequency (%)**			
**< −3**	108 (100)	65 (60.2)	
**-3--2**	0 (0.0)	24 (22.2)	< 0.001
**-2- < −1**	0 (0.0)	14 (13.0)	
**-1 − + 1**	0 (0.0)	5 (4.6)	

The maximum duration of hospital stay among the studied infants of SST was 30 days with a minimum of 4 and a median (IQR) of 9 (3.8) days.

Cox proportional hazards regression model was used to relate the indicator of demographic and clinical data to time to discharge (in days) from hospital after feeding on SST. The only 2 statistical predictors of hazard in the adjusted model were poor appetite and anemia on admission, *p* = 0.017 and 0.033 respectively. The shorter the duration of recovery, the earlier was the discharge.

Hence, the younger the infant the better the outcome on feeding with SST. There was no correlation found between the age and the duration of SST (*r* = 0.028, *p* = 0.777).

A logistic regression model was employed to determine whether the covariates had any effect on the binary divided dependent variable “the outcome”. Among 10 covariates, the only predictor of the outcome was the duration of SST treatment (OR = 1.71, 95% CI = 0.05-0.62, *p* < 0.001) even after adjusting for other covariates (Table 3). Therefore, the longer the duration of treatment (> 10 days) the better was the outcome noted.

**Table 3:** Logistic regression model of clinical predictors of the outcome of treatment of infants with SAM fed on supplementary suckling technique

Independent Covariates	Univariate			Multivariate		
	OR	95% CI	***p***	OR	95% CI	***p***
**Age**	0.9	0.60-1.3	0.519	0.88	0.57-1.35	0.556
**Sex**	1.4	0.52.3.52	0.538	0.99	0.33-2.95	0.987
**Vomiting**	0.71	0.27-1.84	0.476	0.63	0.18-2.18	0.468
**Diarrhea**	0.67	0.25-1.76	0.414	0.68	0.20-2.29	0.531
**Anemia**	1.35	0.36-5.15	0.658	1.50	0.30-7.42	0.618
**Pneumonia**	1.45	0.55-3.84	0.457	1.55	0.51-4.69	0.440
**Seizure**	2.13	0.18-24.61	0.546	2.98	0.17-51.78	0.454
**Weight-for-age z score on admission**	0.86	0.58-1.31	0.860	0.42	0.16-1.08	0.072
**Weight-for-age z score at discharge**	1.10	0.76-1.58	0.628	2.48	0.95-6.44	0.063
**Duration of treatment**	1.71	0.05-0.62	< 0.001*	0.19	0.05-0.76	0.019*

*OR* odd ratio, *CI* confidence interval

## Discussion

There was a high cure rate of infants with SAM feeding on SST with a relatively low case fatality rate of 5.6% and a non-recovery rate of 1.9%. The nutritional indices (weight, weight-for-length Z score and weight for age Z scores) improved on feeding with SST during a median duration of treatment of 9 days. Thus, it was documented that the younger the infant, the better the outcome on feeding with SST and the longer the duration of treatment, the better was the outcome.

This, to the best of our knowledge, is the first study conducted to assess the outcome of SST in infants with SAM in a hospital TFC in a country devastated by conflict. Especially SAM was the result of failure of lactation due to maternal stress in a population with a hazardous combination of factors, driven by conflict and economic decline.

The success rate of SST of SAM infants in this study was 80.6%. Comparatively, Singh et al reported a lower rate (72.7%) however, their sample (44 infants) was comparatively smaller in contrast to this study’s sample. Accordingly, the cure rate in this study was higher than the 72.2% rate of Singh et al and their failure rate was much higher than in this report though, their mortality was lower [16]. Their sample size was the same as in this study however infants were discharged after 5 days of feeding and a follow-up was offered on two weekly intervals. Follow-up information was not recorded in this study. The lower failure rate in this study could be attributed to the relatively larger sample and the commitment to the feeding technique which depended mainly on the breast milk as a natural source and hence successful re-lactation. Moreover, and despite limited resources in Yemen, there was much care and early treatment driven by the application of the guidelines [9]. In a similar report from Niger though with a larger sample, the infants had comparable clinical presentations on admission, comparable similar cure and fatality rate and a lower defaulter rate [17]. The conflict setting in this study’s environment likely hindered similar outcomes when compared to stable communities as in Niger. Nevertheless, the improvement in nutritional indices in both studies did not show any difference but the hospital stay was shorter in this cohort. Though adherence to discharge criteria was strict in this study, mothers demanded early discharge because they had to take care of other children in a conflict setting and endangered homes.

In this cohort, younger children showed a better outcome. In all other studies employing SST as the main feeding procedure which were reviewed, it was quite difficult to find a comparable result due to the lack of literature reports in this aspect [10, 16, 17]. The noticeable response in young children was probably due to the relatively safe environment in the hospital and hence early production of breast milk. Re-lactation was found to be associated with the age of infants; where the relationship showed that the younger the age of the infant at the time of intervention the better was the re-lactation achievement [18].

In contradistinction to Vygen et al., only a duration of hospital stay of more than 10 days was a factor for a better outcome. They reported anorexia, infection and any presenting pathology at admission as risk factors for treatment failure and death [17]. Their sample size (*n* = 632) was much larger than the one in this study and their criteria for discharge is different from the one used in this study among other differences of SST protocol. Long duration of treatment might possibly establish better and independent breastfeeding. Human breastmilk is the best source of nutrients and it also enhance immunity protecting the baby from many illnesses and hence healthy growth and development [19].

This study has many limitations. The study would have yielded better conclusions if a controlled group with infants feeding on RFT was sought. Information of follow-up were not recorded because children were followed in their respective health centers with all due difficulties because of safety issues and displacement. Hence, no reliable information on relapses, readmissions and death therefore, limitation of short and long terms outcomes.

## Conclusion

Supplementary suckling technique for feeding infant with SAM aged under 6 months had a positive impact on anthropometric indices with high cure rate. It was documented in this study that the younger the infant and the longer the duration of treatment, the better was the outcome.

## Supplementary Information

**Additional file 1.**

## Data Availability

The datasets used and/or analyzed during the current study are available from the corresponding author on reasonable request.

## Abbreviations

SAM: Acute severe malnutrition
TFC: Treatment feeding centre
SST: Supplementary sulking technique
WLZ: Weight-for-length z score
