# Supplementary material for: Supplementary suckling technique in infants less than 6 months of age with uncomplicated severe acute malnutrition: a prospective hospital-based study in armed conflict Yemen
Source: BMC Pediatr. 2022 Nov 21;22:671. doi: 10.1186/s12887-022-03745-w (PMC9680131; doi:10.1186/s12887-022-03745-w)
Supplement: Supplementary file 1 — Additional file 1. [file 12887_2022_3745_MOESM1_ESM.docx]

**ANNEX I**

**Effectiveness of Supplementary Suckling Technique in infants less than 6**

**Months of Age with Severe Acute Malnutrition in Aden and Khartoum.**

_Serial No. ………………. Tel. No.: Country:_

**1. PERSONAL DATA:**

1.1 Name:…………………………………………………………………..

1.2 Age group: 1)< 2 months 2) 2-6 months

1.3 Sex:1)Male 2)Female

1.4 Present Residence:1)Aden or (Khartoum) 2)Out of Aden or( Khartoum)

**2. Presence of complications during admission:** Appetite:1) Hunger 2) Normal 3) PoorVomiting:0) No1) Yes Diarrhea:0) No 1) Yes Anemia:0) No1) Yes Clinical Pneumonia:0)No 1)YesSeizure:0)No 1) Yes

**3. Duration of stay in hospital: 1) < 1 wk 2) 1-2 wk 3) > 2 wk**

**4. Outcome on discharge: 1) Cured 2) Defaulted 3) Non-responded4)Died**

**5. PAST HISTORY:**

Previous hospital admission: 0) No 1) Yes if yes specify……….

**6. Vaccination:1)**Up to date 2)Partial 3)Not vaccinated

**7. PSYCOSOCIAL HISTORY OF Family:**

**Father**: Education:1) Illiterate 2) Primary 3) Secondary 4) University

Occupation: **1) Governmental 2) Private 3) Military**

Income per month:1)<100 $2)100-200 $ 3)>200$

**Mother: : Education:** 1) Illiterate 2) Primary 3) Secondary 4) University

**Occupation:1) Governmental 2) Private 3) Military 4) Housewife**

7.1Marital status:1) Married:2)Divorced:3)Widow:4)Others: specify………….

7.2 Length of gestation…….

7.3 Breast feeding:0)No BF 1)Partial BF 2)Exclusive BF

7.4 No. of alivechildren:1)<32) 4-7 3)>7

7.5 Death of children: 0)No 1)Yes if Yes a. Cause of death…….b. No. of deaths…..

7.6 Catastrophe: 0)No 1)Yes If yes specify:…………………….

7.7 Home Displacement: 0)No 1) Yes

7.8 War affection: 0)No1)Yes

7.9 House:1) Owned 2)rented,No. of rooms ( ) water supply0)No 1)Yes , electricity 0)No 1)Yes, Satellite TV0)No 1)Yes

Social class1)Very poor 2)Poor 3)Satisfactory 4) very good

7.10 Birth interval: 1)<12months2)12-24 months 3)> 24 months

7.11 Natal and Post Natal:

7.11.1 Maturity: 1)Term 2)Preterm

7.11.2 No of Births: 1)Singleton: 2)Twins

7.11.3 Birth Weight: 1)Small(<2.5Kg)2) Average(2.5-4.5Kg)3) Large(>4.5Kg)

**8. PHYSICAL EXAMINATION:**

8.1 Anthropometric Examination:

8.1.1 Weight(admission)…..Kg:

8.1.2Lenght(admission)…....cm:

8.1.3 Weight for Lenght:1) <-3 Z score 2) -3-<-2 Zscore 3)-2-<-1 Zscore 4)-1-+1Zscore

8.1.4 MUAC on admission…….Kg:

8.1.5Oedema on admission:1)Not Present 2) Present:

8.1.6 Weight(discharge)…..Kg:

8.1.7Lenght (discharge)……..cm

8.1.8 Weight for Lenght:1) <-3 Z score 2) -3-<-2 Zscore 3)-2-<-1 Zscore 4)-1-+1Zscore

8.1.9 MUAC on discharge….Kg:

8.1.10Oedema on discharge :1)Not Present 2) Present:

8.1.7 H.C. ……….cm:

8.2 Pallor:1) Not pale 2) Pale3) Very pale

8.3 Wasting: 0)No1)Yes

**9. Type of Milk: 1) DF100 with SST 2)F75 with SST**

**10. Date of start SST………………..**

**11. Date of Stop DF100/ F75 for exclusive BF………………..**

**12. INVESTIGATIONS:**

12.1.1Hb …….gm/dl (Before): 1)<7gm/dl 2)7-11 gm/dl 3)>11gm/dl

12.1.2 Hb……..gm/dl (After) : 1) <7gm/dl 2)7-11 gm/dl 3)>11gm/dl

12.2.1 Serum Zinc …μg/dl (Before):1) Low 2) Normal 3) High

12.2.2 Serum Zinc…..μg/dl (After): 1) Low 2) Normal 3) High

12.3.1 Serum Selenium…mg/dl(Before):1) Low 2)Normal3)High

12.3.2 Serum Selenium….mg/dl(After): 1) Low 2) Normal 3) High
